# Human CD4 T Cells From Thymus and Cord Blood Are Convertible Into CD8 T Cells by IL-4

**DOI:** 10.3389/fimmu.2022.834033

**Published:** 2022-02-11

**Authors:** Helena Nunes-Cabaço, Andreia Ramalho-dos-Santos, Ana R. Pires, Leila R. Martins, João T. Barata, Ana E. Sousa

**Affiliations:** Instituto de Medicina Molecular João Lobo Antunes, Faculdade de Medicina, Universidade de Lisboa, Lisbon, Portugal

**Keywords:** CD4+ and CD8+ T cell lineage commitment, IL-4, human thymus, cord blood, innate-like T cells

## Abstract

Commitment to the CD4+ or CD8+ T cell lineages is linked to the acquisition of a functional program broadly defined by helper and cytotoxic properties, respectively. The mechanisms underlying these processes in the human thymus remain largely unclear. Moreover, recent thymic emigrants are thought to have some degree of plasticity, which may be important for the shaping of the immune system and adjustment to specific peripheral needs. We show here that IL-4 induces proliferation-independent *de novo* synthesis of CD8αβ in human CD4 single-positive (SP) thymocytes, generating a stable CD8SP population that features a diverse TCRαβ repertoire, CD4 expression shut-down and ThPOK downregulation. IL-4 also promotes an innate-like program in both CD4SP and CD8SP thymocytes, characterized by Eomes upregulation in the absence of T-bet, in line with its recognized role in the generation of thymic innate-like CD8+ T cells. The clinical relevance of these findings is further supported by the profile of IL-4 production and IL-4 receptor expression that we identified in the human thymus. Importantly, human cord blood CD4+ T cells preserve the ability to generate Eomes+ CD8+ T cells in the presence of IL-4, with implications in neonatal immunity. Our results support a role for IL-4 in the dynamic regulation of human thymocyte plasticity and identify novel strategies to modulate immune responses.

## Introduction

CD4^+^ and CD8^+^ T cell development follow a stringent program in the thymus that ensures highly efficient peripheral responses. This involves validation of TCR specificity together with cytokine signaling, leading to the formation of discrete helper or cytotoxic lineages, respectively. However, many of the mechanisms underlying lineage commitment remain unclear, as well as the degree of cell plasticity. In humans, hematopoietic precursors first acquire CD4 (CD4 immature single positive thymocytes, CD4ISP) and then CD8 expression, generating CD4^+^CD8^+^ double positive (DP) cells that will undergo lineage commitment into helper CD4 single-positive (CD4SP) or cytotoxic CD8SP thymocytes (1). Thymocyte maturation has been discretely classified in terms of the expression of CD69, CD27, CD1a and CD45RA (1–3). Positive selection is associated with CD69 up-regulation at the DP CD3^dim^ to CD3^high^ stages in CD1a-expressing thymocytes. Subsequent CD27 acquisition has been linked to lineage commitment, since DPs expressing CD27 can only differentiate into CD8SP in fetal thymic organ cultures (FTOC), while expression of CD27 on CD4SP thymocytes associates with commitment to the CD4 lineage. Finally, downregulation of CD1a and expression of CD45RA on SP cells mark acquisition of a mature phenotype and potential for thymic egress (1–3).

Mutual antagonistic repression of the transcription factors ThPOK (T-helper-inducing POZ/Krüppel-like factor; also known as cKrox and encoded by the *Zbtb7b* gene, hereafter referred to as *ThPOK*) and the Runx3 member of the Runx family dictates lineage fate in the thymus, as observed in mouse models (4, 5). ThPOK is a crucial regulator of CD4 lineage development that is necessary and sufficient for helper T cell commitment, directing positively selected thymocytes to a CD4 fate regardless of the MHC specificity of their TCR (4–7). Conversely, Runx3 promotes CD8 lineage commitment, namely through *Cd4* silencing (5, 8, 9). In addition, the zinc-finger transcription factor GATA-3 is induced by TCR signal strength and its requirement appears to precede ThPOK in CD4 T cell development (10, 11). Another transcription factor termed Mazr (Myc-associated zinc finger-related factor) has been shown to contribute to CD8 lineage commitment through repression of ThPOK expression, although it may also regulate CD4 commitment (12).

Once the helper vs. cytotoxic lineage fate is established, mature SP thymocytes shut down the expression of the other coreceptor. The CD8-CD4 lineage dichotomy persists in peripheral mature T cells, as ThPOK continues to suppress the cytotoxic fate in CD4 T cells (13). Nevertheless, some degree of plasticity is still evident in CD4 T cells upon chronic or strong stimulation, suggesting that CD8 repression may be reversible in particular microenvironments, as reported in the gut mucosa (14, 15).

Studies in mice have established a kinetic lineage commitment model whereby developing TCRαβ^+^ CD4^+^CD8^+^ DP thymocytes undergoing positive selection initially terminate CD8 transcription in order to test their TCR specificity. At this point, a long and persistent TCR signaling leads to CD4SP cell commitment, whereas rapid loss of TCR engagement together with suitable cytokine signaling result in reactivation of *Cd8* expression and cytotoxic CD8SP cell generation (5, 16). IL-7 has been shown to be the main cytokine involved in this process, termed “coreceptor reversal”, although the involvement of other γ-chain (γc) cytokines has also been reported (17).

Interleukin-4 (IL-4), a short four-helix bundle peptide member of the common γc receptor cytokine family, is a pleiotropic cytokine produced mainly by lymphocytes, namely Th2, γδ T, NKT and T follicular helper cells, as well as by mast cells, basophils and eosinophils (18). IL-4 regulates different processes in multiple cell types. In addition to its role in B cell differentiation, it promotes Th2 by inhibiting Th1 and Th17 fates in T cells (19). IL-4 is also involved in CD8 T cell function, as it promotes the proliferation and cytotoxic activity of CD8 T cells (20–22) or can act as a negative regulator of CD8 T cell responses (23). In the thymus, IL-4 is required for the development of innate-like CD8 T cells, which feature phenotypical and functional traits of memory T cells in the absence of foreign antigen stimulation (24). Innate-like CD8 T cells express high levels of CD44, CXCR3, CD122 and Eomes but not T-bet, and are rapid IFN-γ producers upon activation (24). Their development is highly dependent on IL-4 production by cells expressing the promyelocytic leukemia zinc finger protein (PLZF), which include invariant NKT (iNKT) cells in mice and CD4 T cells that resulted from MHC class II-dependent thymocyte-thymocyte interactions (T-T CD4) in humans (25). Eomes^+^ innate-like CD8 T cells were identified in human fetal thymuses and in cord blood, but their thymic expression was reported to rapidly decrease after birth until 3 months of age (26, 27). Importantly, responsiveness to IL-4 during murine CD8 T cell development and homeostasis may alter their functional reactivity and response to pathogens (28).

Here, we found that IL-4 upregulates CD8αβ and downregulates CD4 and ThPOK in human CD4SP thymocytes, leading to their conversion into CD8 T cells. Moreover, a population of innate-like cells is induced, as defined by the expression of Eomes and CXCR3 in the absence of T-bet. The effects of IL-4 extended to CD4 T cells from cord blood and progressively declined in the periphery with age and cell differentiation stage, indicating a preferential impact in the neonatal period.

## Materials and Methods

### Human Samples

Human thymic specimens were from newborns to 14-month-old children undergoing routine thymectomy performed during corrective cardiac surgery at Hospital de Santa Cruz, Carnaxide, Portugal. Cord blood was collected at the Department of Obstetrics of Hospital de Santa Maria/Centro Hospitalar Universitário Lisboa Norte (CHULN), Lisbon, Portugal. Pediatric tonsils and paired blood were from 4- to 5-year-old children and were obtained from routine pediatric tonsillectomy at the ENT department of Hospital de Santa Maria/CHULN, Lisbon, Portugal. All samples were collected after parent’s/tutor’s written informed consent. The study was approved by the Ethical Boards of Hospital de Santa Cruz, Faculty of Medicine of the University of Lisbon and CHULN.

### Sample Processing

Total thymocytes and tonsillar cells were recovered through tissue dispersion using a 70μM filter and a syringe plunger. Mononuclear cells were separated from tissue suspension or from heparinized cord, pediatric or adult blood using Ficoll-Paque PLUS (GE Healthcare) density gradient. The cord blood mononuclear cell suspension was further incubated with BD FACS Lysing Solution (BD Biosciences) for red blood cell lysis.

### Cell Separation, Labeling and Culture

CD4SP or CD8SP cells were sorted (purity>98%) from total thymocytes as CD3^high^CD4^+^CD8^neg^CD25^neg^TCRγδ^neg^ or CD3^high^CD4^neg^CD8^+^CD25^neg^TCRγδ^neg^ cells, respectively, using a FACSAria High-Speed Cell Sorter (BD Biosciences). CD4+ T cells were FACS-purified from blood or tonsils as CD3^high^CD4^+^CD8^neg^ (purity>97%). Anti-human monoclonal antibodies (mAbs) used were: CD3 (UCHT1), CD4 (RPA-T4), CD8α (RPA-T8), CD25 (2A3) and TCRγδ (B1.1) from eBioscience/Thermo Fisher Scientific or BD Biosciences. Where indicated, cells were labeled with 1μM CellTrace Violet (CTV; Thermo Fisher Scientific) for 20 minutes at room temperature. Sorted populations were resuspended at 1-2x10^6^ cells/mL in complete medium (RPMI1640 with 10% fetal bovine serum (FBS), 2mM L-glutamine and 50U/mL penicillin/streptomycin, all from GIBCO/Invitrogen) and cultured in the presence or absence of IL-2 (20U/mL), IL-4 (20ng/mL; R&D or Peprotech) or IL-7 (10ng/mL, R&D) for 6 or 7 days at 37°C and 5% CO_2_. Where mentioned, an anti-IL-4 neutralizing antibody (MP4-25D2, eBioscience) was added to the IL-4 culture at the specified concentrations. For gene expression analysis, thymocyte populations were FACS-sorted as TN (Lin^neg^CD3^neg^CD4^neg^CD8^neg^CD34^+^), CD4ISP (Lin^neg^CD3^neg^CD4^+^CD8^neg^), DP (Lin^neg^CD4^+^CD8^+^), CD4SP (Lin^neg^CD3^high^CD4^+^CD8^neg^) or CD8SP (Lin^neg^CD3^high^CD4^neg^CD8^+^) cells using additional anti-human mAbs such as CD11c (3.9), CD14 (61D3), CD16 (eBioCB16), CD19 (HIB19), CD20 (2H7), CD34 and CD123 (6H6) from eBioscience/Thermo Fisher Scientific. Sorted cells were frozen as dry pellets and stored at -80°C until further use.

### Flow Cytometry Staining and Analysis

Surface staining was performed for 20 minutes at room temperature and always included Fixable Viability Dye (eBioscience) for dead cell exclusion. For intracellular analysis thymocytes were fixed, permeabilized and stained using the Transcription Factor Staining Buffer Set (eBioscience), as per manufacturer’s instructions. Other anti-human mAbs used included: CD8β (SIDI8BEE), CD27 (O323), CD45RA (HI100), CD44 (IM7), CD69 (FN50), CXCR3 (1C6), Eomes (WD1928), T-bet (eBio4B10), ThPOK (ZFP-67), Runx1 (RXDMC), Runx3 (R3-5G4) and Ki67 (B56) from eBioscience/Thermo Fisher Scientific, BD Biosciences or R&D Systems. Cells were acquired in a LSRFortessa (BD Biosciences) cytometer and data was analyzed using FlowJo v10 (FlowJo, BD). For the visualization of high-dimensional data through the t-distributed stochastic neighbor embedding (t-SNE) dimensionality reduction technique, similar number of live, single cells from each condition were concatenated. tSNE parameters were set to 1,000 iterations, perplexity 30 and learning rate 4200, and based on appropriate markers.

### Thymic Organ Cultures (TOCs)

Thymic tissue blocks (1-2 mm diameter) were placed on Isopore membranes (Millipore) in a 6 well plate containing 2 mL of TOC medium (complete medium but with 15% FBS and addition of 10mM HEPES, 1mM sodium pyruvate and 1% MEM non-essential aminoacids, all from GIBCO/Invitrogen), without (control) or with addition of IL-4 (10 ng/ml), and cultured at 37°C and 5% CO_2_. A third of the media was replaced every 2-3 days. On day 6 post-infection TOCs were mashed and thymocytes were stained and analyzed by flow cytometry.

### Functional Studies

CD4SP thymocytes were cultured for 7 days with IL-4 and subsequently FASC-sorted into tCD4SP (CD4^+^CD8^neg^), iDP (CD4^+^CD8^+^) and iCD8SP (CD4^neg^CD8^+^) populations. Sorted cells were either stimulated with 50ng/ml PMA and 500ng/ml ionomycin in the presence of 10μg/mL brefeldin for 4h at 37°C and 5% CO_2_, or they were further TCR-stimulated using Dynabeads Human T-activator CD3/CD28 (Thermo Fisher Scientific) for 5 days before the PMA/ionomycin protocol. The same stimulation procedures were applied to e*x vivo* CD4SP and CD8SP cells, as controls. After stimulation cells were surface-stained, fixed with formaldehyde 2%, permeabilized with 0,5% saponin in PBS/BSA/azide and incubated with intracellular antibodies, including anti-IFN-γ (4S.B3) and anti-TNF-α (MAb11) from Thermo Fisher Scientific, before acquisition in an LSRFortessa cytometer.

### IL-4 Production by Thymocytes *Ex Vivo*

Total thymocytes were incubated with 10μg/mL brefeldin for 4h at 37°C and 5% CO_2_, surface-stained, fixed with formaldehyde 2% and permeabilized with 0,5% saponin before incubation with intracellular antibodies. Anti-human mAbs used included: CD16 (eBioCB16), CD56 (NCAM), HLA-DR (L243) and anti-IL-4 (8D4-8), all from Thermo Fisher Scientific, Biolegend or BD Biosciences. The following live (FVD^neg^), singlet thymocyte populations were analyzed: γδ T cells (CD3^high^TCRγδ^+^); CD16^+^/CD56^+^ cells (TCRγδ^neg^CD16^+^/CD56^+^); and within TCRγδ^neg^ CD16^neg^ CD56^neg^ cells: TN (CD3^neg^CD4^neg^CD8^neg^), CD4ISP (CD3^neg^CD4^+^CD8^neg^), DP CD3^low^ (CD3^low^CD4^+^CD8^+^), DP CD3^high^ (CD3^high^CD4^+^CD8^+^), CD4SP (CD3^high^CD4^+^CD8^neg^), CD8SP (CD3^high^CD4^neg^CD8^+^) and HLA-DR^+^ cells.

### Gene Expression Quantification

100 ng of total RNA purified with ZR-Duet DNA/RNA MiniPrep Kit (Zymo Research) were used to synthesize cDNA using random primers and Superscript III (Invitrogen). For the quantification of *IL4RA* and *ACTB* (reference gene), cDNA pre-amplification and real-time PCR (with TaqMan^®^ PreAmp and Taqman^®^ Gene Expression Master Mix, respectively; Applied Biosystems) were performed using gene expression assays (*IL4RA*: Hs00166237_m1; *ACTB*: Hs99999903_m1; Thermo Fisher Scientific). *EOMES*, *TBX21* (T-bet), *MAZR*, *ETS1* and *IL13RA* were quantified using predesigned KiCqStart^®^ SYBR^®^ Green Primers (Sigma-Aldrich) and Power SYBR^®^ Green Master Mix (Thermo Fisher Scientific). Quantification of *CD4*, *CD8*, *ThPOK (ZBTB7B*), *GATA3*, *RUNX1*, *RUNX2*, *RUNX3* and *ACTB* was also performed with SYBR^®^ Green using Applied Biosystems ViiA7 Real-Time PCR System, and the following primers were used (Forward/Reverse): *CD4*: TGCCTCAGTATGCTGGCTCT/GAGACCTTTGCCTCCTTGTTC; *CD8*: TCCTCCTATACCTCTCCCAAAAC/GGAAGACCGGCACGAAGTG; *ZBTB7B*: GTCCCCAGAGCTACGAACC/AGCTTAGGTAGGCCATCAGGT; *GATA3*: GCCCCTCATTAAGCCCAAG/TTGTGGTGGTCTGACAGTTCG; *RUNX1*: ACTATCCAGGCGCCTTCACCTACT/TAGTACAGGTGGTAGGAGGGCGAG; *RUNX2*: ACGAATGCACTATCCAGCCACCTT/ATATGGAGTGCTGCTGGTCTGGAA; *RUNX3*: AGGCAATGACGAGAACTACTCC/CGAAGGTCGTTGAACCTGG; *ACTB*: CTGGCACCCAGCACAATG/GCCGATCCACACGGAGTACT.

### Immunohistochemistry

Formalin-fixed thymic tissue was embedded in paraffin and cut into 3μm sections (Minot Microtome Leica RM2145). Epitope-retrieval was performed at pH9 (Leica Biosystems buffer) for 15min using a microwave (800W). Samples were stained with antibodies against human IL-4, incubated with a peroxidase/DAB detection system (EnVision, Dako) and counterstained with Harris’ hematoxylin (BioOptica). Images were acquired using a Leica DM2500 microscope.

### Mouse Studies

CD4SP cells were sorted from the mashed thymus of 9-day-old or 5-week-old C57BL/6 mice as TCRβ^high^CD4^+^CD8^neg^ (purity>98%) using a BD FACSAria High-Speed Cell Sorter, cultured with murine IL-4 (20 ng/ml; Peprotech) or IL-7 (10 ng/ml) for 6 days as described above and analyzed by flow cytometry.

### Statistics

Statistical analysis was performed using GraphPad Prism v7 (GraphPad Software Inc.) and results are presented as mean±SD. Two-sample data were compared using Wilcoxon-matched pairs test. Data from more than two samples were compared using Friedman test or Kruskal-Wallis test with Dunn’s multiple comparison post-test, considering CD4SP thymocytes *ex vivo* as control values whenever cells were cultured with cytokines. Only statistically significant data (P value<0.05) is presented.

## Results

### IL-4 Induces the Generation of Mature DP and CD8SP Thymocytes From Human CD4SP Thymocytes

**Figure 1:**
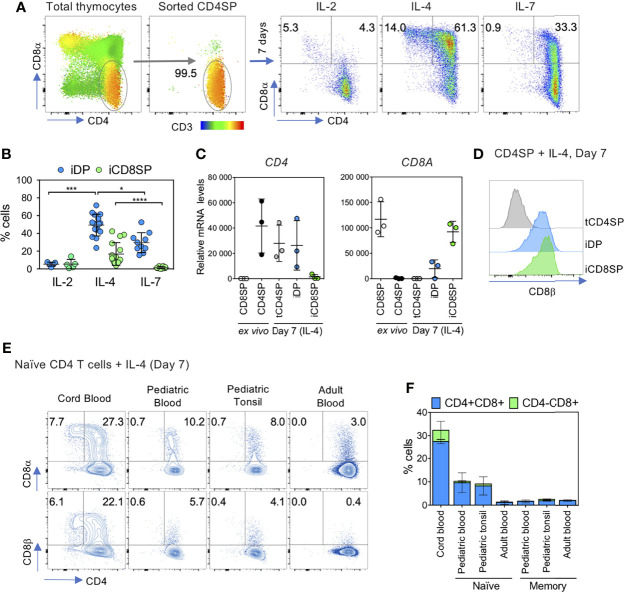
IL-4 induces the upregulation of CD8αβ on human CD4SP thymocytes and in peripheral CD4^+^ T cells. **(A)** CD4SP thymocytes, sorted as CD3^high^CD4^+^CD8^neg^CD25^neg^ TCRγδ^neg^, were incubated with IL-2, IL-4 or IL-7 for 7 days and CD8α expression was assessed at the end of the culture. CD3 expression is presented as an heatmap overlay on initial populations. **(B)** Frequency of cytokine-induced double-positive (iDP) and CD8 single-positive (iCD8SP) thymocytes after 7 days of culture with IL-2, IL-4 or IL-7. Each dot represents a thymus. Statistics was only performed within the same subset (iDP or iCD8SP). **(C)** *CD4* and *CD8A* mRNA expression in CD4SP and CD8SP thymocytes *ex vivo* and in sorted populations 7 days after IL-4 culture. tCD4SP: cytokine-treated CD4 single-positive thymocytes. **(D)** Expression of CD8β on tCD4SP, iDP and iCD8SP generated from incubation of CD4SP thymocytes with IL-4 for 7 days. **(E)** Expression of CD8α and CD8β on sorted naïve CD4^+^ T cells from cord, pediatric or adult blood and pediatric tonsil after a 7-day culture with IL-4 (left dot plots) and **(F)** quantification of iDP and iCD8SP cells generated from naïve and memory T cells (right panel; n=2/3). Results in graphs are presented as mean ± SD. *p < 0.05, ***p < 0.001, ****p < 0.0001.

We noticed a marked upregulation of CD8αβ on human CD4SP thymocytes when cultured in the presence of IL-2, IL-4 and IL-7 to facilitate cell survival in studies of HIV infection (29). This prompted us to investigate which cytokine was responsible for this effect by culturing CD4SP thymocytes, depleted of CD25^+^ and TCRγδ^+^ cells, in the presence of each cytokine (**Figure 1A**). IL-4 displayed the strongest ability to promote CD8αβ expression, resulting in the generation of induced double positive DP (iDP) and CD8SP (iCD8SP) cells (**Figures 1A, B** and **Figure S1A**), irrespective of the concentration used (**Figure S1B**; above 10 ng/ml for iDP cells), the time-point analyzed (**Figure S1C**) or the age of the thymic donor (**Figure S1D**). Both iDP and iCD8SP cells maintained high CD3 levels, in agreement with preservation of a mature phenotype (**Figure S1E**). In addition, neither IL-13 nor IL-15, whose receptors share IL-4Rα and γc subunits with the IL-4 receptor, respectively, were able to induce CD8 expression on CD4SP thymocytes (**Figure S1F**). Notably, addition of IL-4 to thymic organ cultures (TOCs) markedly reduced the CD4SP/CD8SP ratio (**Figure S1G**), supporting its impact also in the tissue microenvironment. Moreover, the addition of an anti-IL-4 neutralizing antibody to the IL-4 culture decreased the upregulation of CD8 and the generation of iCD8SP cells (**Figure S1H**).

Importantly, the increase in CD8α protein in iCD8SP cells was paralleled by *CD8A* upregulation and the shutdown of *CD4* at the transcriptional level (**Figure 1C**). *CD8A* upregulation was already noticeable at 4h post-IL-4 culture (mean ± SD fold-change 1,9 ± 0,6, n=3). While CD4 is a monomeric type I transmembrane glycoprotein, CD8 exists as two isoforms, CD8αα and CD8αβ, expressed on different cell types and with different functions. Conventional CD8SP thymocytes express CD8αβ, which has been associated with greater signaling ability than CD8αα (30). Mice lacking CD8β are devoid of peripheral mature CD8 T cells (31). Thus, we examined the composition of the CD8 molecule upregulated in CD4SP thymocytes after IL-4 exposure. Indeed, we confirmed that iDP and iCD8SP expressed the CD8β subunit, and thus consisted of the CD8αβ heterodimer (**Figure 1D** and **Figure S1E**).

Interestingly, IL-4 was able to induce CD8 expression in both CD27^neg^ and CD27^+^ CD4SP thymocytes (**Figure S1I**). Moreover, although the potential to respond to IL-4 declined with CD4SP maturation, as assessed by the expression of CD27 and CD45RA, we observed the generation of iCD8SP cells even from CD27^+^CD45RA^+^ thymocytes (**Figure S1J**).

We then asked whether IL-4 was also able to induce CD8 expression on murine CD4SP thymocytes *in vitro*. In comparison to the human thymus, we found very low CD8 upregulation and reduced generation of iDP and iCD8SP cells upon exposure of mouse TCRβ^high^ CD4SP thymocytes to IL-4 (**Figure S1K**). Of note, in the murine system equivalent to the human system used both IL-4 and IL-7 had low ability to induce CD8 expression on CD4SP thymocytes (**Figure S1K**).

Next, we investigated the ability of IL-4 to upregulate CD8 expression on peripheral CD4 T cells at different ages and differentiation stages, including: i) total CD4 T cells from cord blood; ii) naïve or memory CD4 T cells from tonsil or paired pediatric blood; and iii) naïve or memory CD4 T cells from adult blood. Cord blood CD4 T cells displayed the highest ability to increase CD8αβ expression and to generate CD8αβ^+^CD4^neg^ T cells upon exposure to IL-4 (**Figures 1E, F**). Moreover, the potential for IL-4-induced CD8αβ upregulation decreased both with age (cord blood>pediatric blood>adult blood), and differentiation stage (naïve>memory), with memory CD4 T cells of any source showing a negligible capacity to increase CD8 expression upon IL-4 culture (**Figures 1E, F**).

In conclusion, we found that IL-4 was able to induce coreceptor reversal in human CD4SP thymocytes, generating iDP and iCD8SP thymocytes with a mature phenotype. Importantly, our observation that IL-4 induced the generation of CD8αβ^+^CD4^neg^ T cells from cord blood CD4 T cells extends the results obtained in the thymus to the periphery.

### CD4SP Thymocytes Express IL-4Rα and Produce High Levels of IL-4

**Figure 2:**
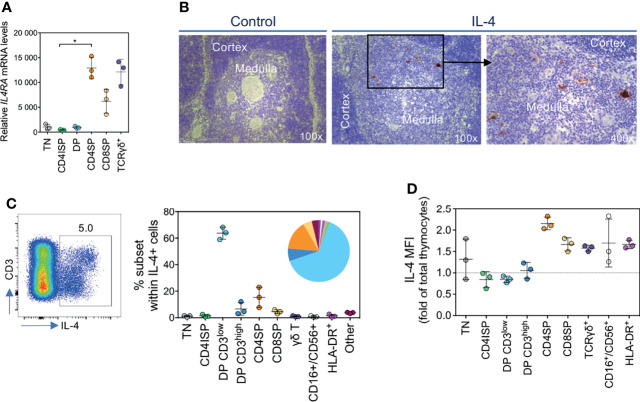
CD4SP thymocytes express IL-4Rα and produce high levels of IL-4. **(A)** *IL4RA* mRNA levels in developing T cells. TN: triple-negative; CD4ISP: CD4 immature single-positive; DP: double-positive; CD4SP and CD8SP: CD4^+^ or CD8^+^ single-positive. **(B)** Analysis of IL-4 expression in the human thymus by immunohistochemistry. **(C)** Flow cytometric analysis of IL-4-producing thymic populations, as frequency of total IL-4 production. **(D)** Median fluorescence intensity (MFI) of IL-4^+^ cells within each subset analyzed. Results in graphs are presented as mean ± SD. *p < 0.05.

The strong direct effect of IL-4 on CD4SP thymocytes indicates that this subset expresses the IL-4 receptor. Indeed, the *ex vivo* analysis of different thymocyte subsets revealed that CD4SP thymocytes expressed high levels of *IL4RA* mRNA, comparable to the expression observed on γδ T cells (**Figure 2A**). Of note, we could not detect *IL13RA* mRNA expression in CD4SP thymocytes, in agreement with the lack of an effect of IL-13 on these cells.

Moreover, in support of a physiological role of IL-4 on CD4SP thymocytes, we found its expression in the human thymus, mainly in the medulla, by immunohistochemistry (**Figure 2B**). Flow cytometry analysis confirmed the existence of a clear population of IL-4-producing thymocytes, even in the absence of stimulation (mean±SD 5.7±2.4% of total thymocytes, n=3; **Figure 2C** and **Figure S2A**). Although the largest contributors to the IL-4 pool were DP CD3^low^ cells, the amount of cytokine on a per cell basis in this population was relatively low (**Figure 2D**). Interestingly, CD4SP thymocytes not only represented a high proportion of the IL-4-producing thymocytes, but also featured the highest levels of IL-4 production at the single-cell level (**Figures 2C, D**). Of note, we found sustained thymic IL-4 production up to 14 months of age, the age of the oldest child submitted to reconstructive cardiac surgery during the study period (**Figure S2B**).

Overall, we showed that CD4SP thymocytes express high levels of IL-4Rα and that IL-4 is mainly produced in the thymic medulla by several types of cells after cells, including CD4SP thymocytes themselves. Our data thus support a possible physiological role of IL-4 in human CD8SP thymocyte development from CD4SP thymocytes.

### iCD8SP Thymocytes Are Stable and Diverse

**Figure 3:**
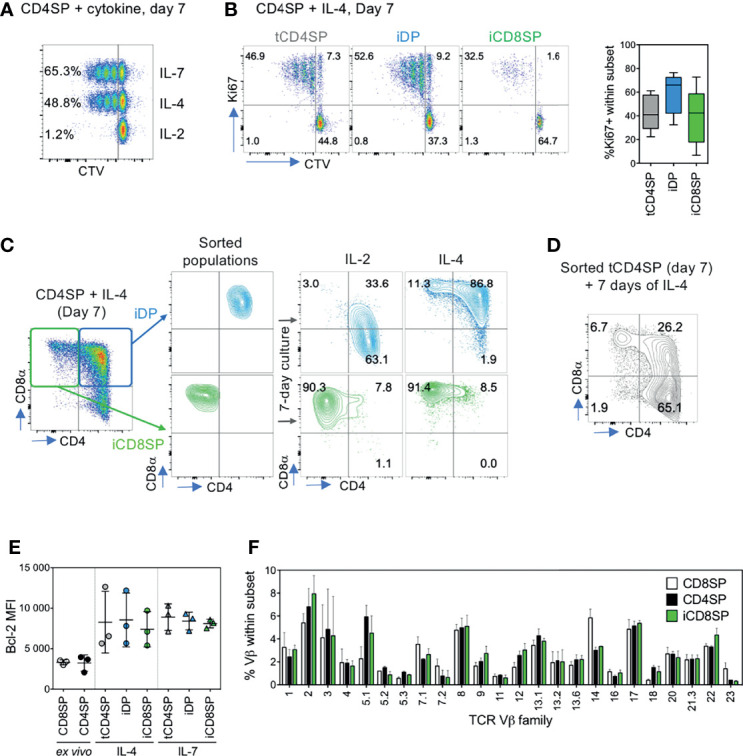
iCD8SP cells generated from CD4SP thymocytes in the presence of IL-4 are stable and diverse. **(A)** Proliferation of CD4SP thymocytes, sorted as CD3^high^CD4^+^CD8^neg^CD25^neg^ TCRγδ^neg^ cells, in response to IL-2, IL-4 and IL-7, as measured by Cell Trace Violet (CTV) dilution. Frequency of CTV^neg^ cells is presented in each population, and data are representative of 3 experiments. **(B)** Proliferation of tCD4SP, iDP and iCD8SP thymocytes in response to IL-4, as measured by CTV dilution or Ki67 frequency (graph: n=5). **(C)** Stability of IL-4-induced CD8 expression, as assessed by sorting iDP and iCD8SP populations on day 7 of IL-4 culture and either maintaining cells in IL-4 or switching them to IL-2 for 7 more days. **(D)** CD8 induction in CD4^+^CD8^neg^ (tCD4SP) cells sorted 7 days after IL-4 culture of CD4SP thymocytes and cultured in the presence of IL-4 for 7 additional days. **(E)** Bcl-2 median fluorescence intensity (MFI) of different populations before and after exposure to IL-4 or IL-7. **(F)** TCR Vβ repertoire of CD8SP, CD4SP and IL-4-induced iCD8SP thymocytes (n=3). Results in graphs are presented as mean ± SD.

Next, we evaluated whether the IL-4-mediated CD8 upregulation and iCD8SP generation required cell proliferation. In order to track the proliferative history of cells in culture, sorted CD25^neg^ TCRγδ^neg^ CD4SP thymocytes were labeled with CTV prior to culturing with IL-4, IL-7 or IL-2 (**Figure 3A**). Both IL-4 and IL-7 induced cell proliferation, resulting in increased cell numbers in the well (mean±SD fold change after 7-day culture: 1,52±0,70 vs 2,25±0,52 for IL-4 and IL-7, respectively; n=4). Robust proliferation was also observed in all subsets identified upon 7-day culture with IL-4, as assessed by both CTV labeling and Ki67 staining (**Figure 3B**). Nevertheless, we found that a large fraction of iCD8SP thymocytes retained high CTV levels and did not express Ki67, and thus did not divide or enter cell cycle (**Figure 3B**), supporting that IL-4-induced coreceptor reversal of CD4SP thymocytes can occur in the absence of cell division. This observation further excluded the possibility of iDP and iCD8SP cells being solely derived from the proliferation of contaminating CD8^+^ cells present at the onset of the culture. Similarly, cell division did not necessarily result in CD8 expression.

We then investigated whether the iCD8SP cells generated from IL-4-treated CD4SP thymocytes (tCD4SP) were stable upon IL-4 removal from the culture. To this end, we sorted IL-4-induced iCD8SP thymocytes, as well as tCD4SP and iDP for comparison, and either replaced IL-4 by IL-2 or, as a control, maintained the culture supplemented with IL-4. Whereas the loss of CD8 expression in iDP cells upon IL-4 removal suggested a transient nature of this population, iCD8SP cells preserved their phenotype in the absence of IL-4, indicating that effective coreceptor reversal of CD4SP thymocytes was stable (**Figure 3C**). Notably, further exposure to IL-4 continued to promote the generation of iCD8SP thymocytes from tCD4SP (**Figure 3D**). Bcl-2 was upregulated upon IL-4 exposure in all populations, likely promoting cell survival, and the levels attained were similar to those observed upon IL-7 culture (**Figure 3E**).

Having established the stability of IL-4-induced iCD8SP cells, we asked whether they bared a diverse TCR repertoire. Comparison of the TCR Vβ family profile of iCD8SP thymocytes with *ex vivo* CD4SP and CD8SP thymocytes revealed that iCD8SP cells indeed present a diverse TCR Vβ repertoire, arguing against their emergence from selective populations within CD4SP thymocytes (**Figure 3F**).

Overall, we found that IL-4 is able to induce coreceptor reversal in human CD4SP thymocytes in a proliferation-independent manner, generating a stable and diverse population of iCD8SP thymocytes.

### IL-4 Induces an Innate-Like Program in Both CD4SP and CD8SP Thymocytes

**Figure 4:**
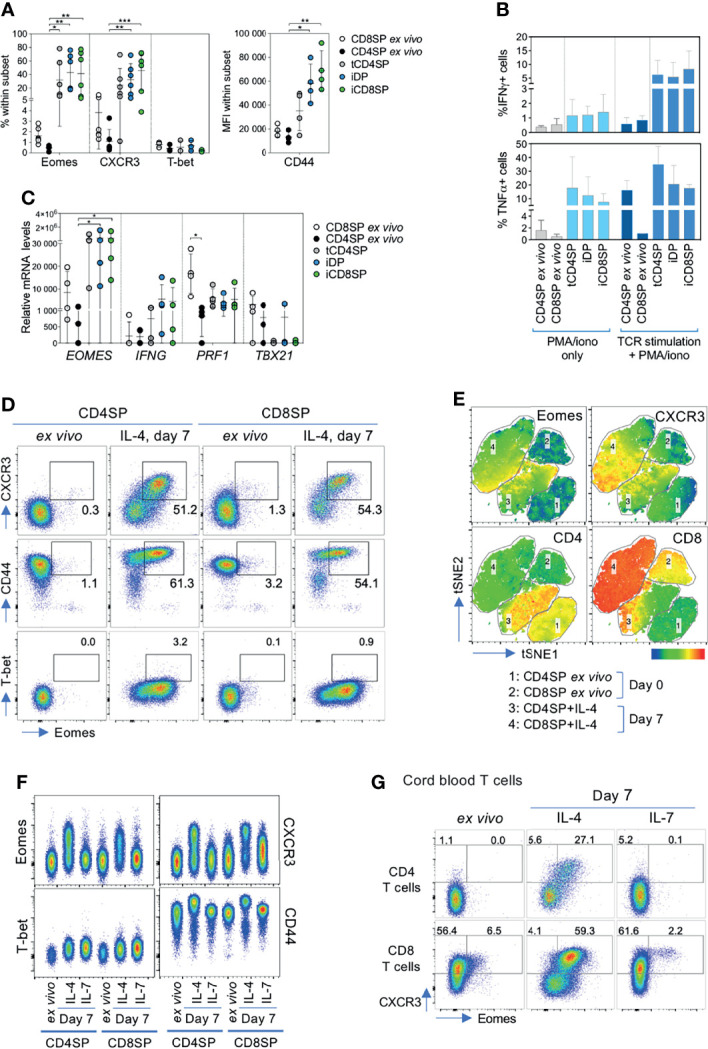
IL-4 induces an innate phenotype on CD4SP thymocytes and cord blood CD4^+^ T cells. **(A)** Frequency of Eomes, CXCR3 and T-bet and MFI of CD44 within CD4SP or CD8SP thymocytes *ex vivo,* or tCD4SP, iDP and iCD8SP cells 7 days after exposure to IL-4. **(B)** Frequency of IFN-γ^+^ and TNF-α^+^ cells within *ex vivo* CD4SP and CD8SP thymocytes or cells treated with IL-4 upon stimulation with PMA/ionomycin, both with or without TCR stimulation for 5 days (n=3-5). **(C)** Transcriptional expression of *EOMES*, *IFNG*, *PRF1* and *TBX21* in CD4SP and CD8SP cells *ex vivo* or after IL-4 culture. **(D)** Representative dot plots of Eomes, CXCR3, CD44 and T-bet expression in CD4SP and CD8SP thymocytes *ex vivo* and after 7 days of culture with IL-4. **(E)** tSNE clustering analysis of the concatenated flow cytometry data of *ex vivo* and IL-4-exposed CD4SP and CD8SP cells, based on the following markers: CD3, CD4, CD8α, CD8β, Eomes and CXCR3. Eomes, CXCR3, CD4 and CD8 expression are presented as heatmap overlays over the populations analyzed. **(F)** Representative dot plots of innate-related markers in CD4SP and CD8SP thymocytes *ex vivo* and after 7 days of culture with IL-4 or IL-7. **(G)** Eomes and CXCR3 expression in cord blood CD4^+^ or CD8^+^ T cells *ex vivo* or cultured with IL-4 or IL-7 for 7 days. Results in graphs are presented as mean ± SD. *p < 0.05, **p < 0.01, ***p < 0.001, ***p<0.0001.

Given the central role of IL-4 in the development of innate CD8 T cells we hypothesized that this cytokine could be inducing an innate-like CD8SP phenotype on human CD4SP thymocytes. Indeed, we found an upregulation of Eomes in CD4SP thymocytes cultured with IL-4 in both isolated cells (**Figure 4A** and **Figure S3A**) and TOCs (**Figure S3B**). Our results also showed an increase in the expression of CXCR3, CD44, IFN-γ, TNF-α, perforin and granzyme B, but not of T-bet (**Figures 4A–C** and **Figure S3C**).

The innate-like program induced by IL-4 in CD4SP thymocytes was observed in all the populations generated, independently of CD8 up-regulation (**Figures 4A–C** and **Figure S3A**). We further confirmed the lack of correlation between Eomes and CD8 expression in CD4SP cultured with IL-4 at day 7 (p=0.102, Spearman r=0,77, n=6). Moreover, the innate-like profile induced by IL-4 on CD4SP cells was comparable to that observed in IL-4-treated CD8SP thymocytes (**Figure 4D** and **Figure S3D**). tSNE clustering of the concatenated flow cytometry data of *ex vivo* and IL-4-exposed CD4SP and CD8SP cells supported the similarity of the populations after IL-4 treatment, as confirmed by their similar upregulation of Eomes and CXCR3 (**Figure 4E**). In addition, tSNE analysis supported the absence of a direct association between the expression of CD8 and innate-like markers (**Figure 4E**). Importantly, this innate-like profile was not observed upon culture with IL-7 (**Figure 4F**). Moreover, the unique ability of IL-4 to induce an innate-like program extended both to CD4 and CD8 T cells in human cord blood (**Figure 4G**).

Overall, our results revealed the potential of IL-4 to induce an innate-like phenotype both in human CD4SP and CD8SP thymocytes, as well as in cord blood T cells, irrespective of CD8 upregulation. These data thus confirm that the IL-4-induced iCD8SP population includes cells with an innate-like profile.

### IL-4 Induction of iCD8SP Cells Is Linked to ThPOK Downregulation

**Figure 5:**
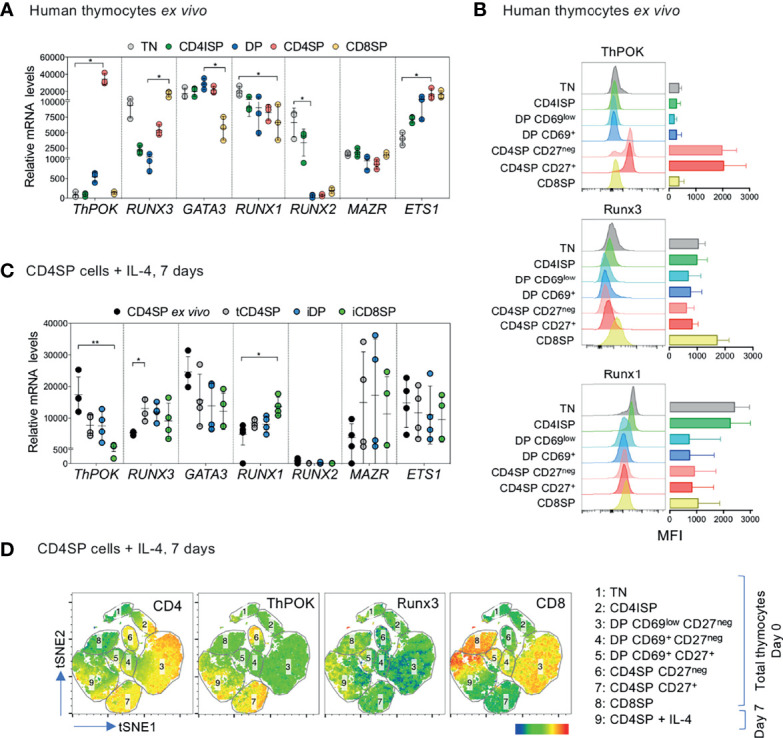
IL-4-induced iCD8SP thymocytes have downregulated ThPOK mRNA and protein. **(A)** Expression of *ThPOK* (*ZBTB7B*), *RUNX3, GATA3, RUNX1, RUNX2, MAZR* and *ETS1* mRNA levels in sorted *ex vivo* human thymocyte populations. TN: triple-negative; CD4ISP: CD4 immature single-positive; DP: double-positive; CD4SP and CD8SP: CD4^+^ or CD8^+^ single-positive. **(B)** Median fluorescence intensity (MFI) of ThPOK, Runx3 and Runx1 expression during human T cell development (n=3). **(C)** *ThPOK* (*ZBTB7B*), *RUNX3, GATA3, RUNX1, RUNX2, MAZR* and *ETS1* mRNA levels in CD4SP thymocytes *ex vivo* and in sorted tCD4SP, iDP and iCD8SP populations after IL-4 treatment. **(D)** tSNE dimensional reduction analysis of concatenated flow cytometry results (total thymocytes and IL-4-treated CD4SP thymocytes), based on the following markers: CD3, CD4, CD8α, CD69, CD27, CD45RA, ThPOK and Runx3. The expression of CD4, ThPOK, Runx3 and CD8 are presented as overlays over the populations analyzed. Results in graphs are presented as mean ± SD. *p < 0.05, **p < 0.01.

To characterize the mechanisms by which IL-4 induces CD8 expression in CD4SP thymocytes, we first determined the mRNA levels of several transcription factors relevant for cell-fate choice, namely *ThPOK* and *GATA3* (CD4 lineage promoting factors), *RUNX3*, *MAZR* and *ETS1* (CD8 lineage-associated factors), as well as *RUNX1* and *RUNX2* (described as main players in early stages of human T cell development) (4, 5). Analysis of the main thymocyte developmental stages confirmed that *ThPOK* and *RUNX3* levels were highest in CD4SP and CD8SP thymocytes, respectively (**Figure 5A**). Expression of other *RUNX* members was increased during early thymocyte development, while Runx3-promoting factor *ETS1* was upregulated in mature subsets but did not present a direct association with the CD8 lineage (**Figure 5A**). CD8SP thymocytes expressed lower *GATA3*, but higher MAZR levels than CD4SP cells (**Figure 5A**).

DP and CD4SP thymocytes were further subdivided according to their expression of the positive selection marker CD69 and the maturation marker CD27, respectively, to assess protein expression of ThPOK, Runx3 and Runx1. Our results confirmed the association of ThPOK with the CD4 lineage, of Runx3 with the CD8 lineage, and of Runx1 with early stages of development (**Figure 5B** and **Figure S4A**).

Next, we evaluated the levels of transcription factors in IL-4-induced populations upon 7-day culture of CD4SP thymocytes both at the transcriptional (**Figure 5C**) and protein (**Figure 5D** and **Figure S4B**) levels. We observed a marked reduction of *ThPOK* levels in iCD8SP thymocytes, indicating the repression of the CD4 transcriptional program. Notably, iDP thymocytes were similar to tCD4SP thymocytes and did not present the same decrease in *ThPOK* mRNA levels as iCD8SP, which, together with their loss of iDP phenotype upon IL-4 removal (**Figure 3C**), further support the transitory nature of the iDP population. We found a minor IL-4-dependent increase in *RUNX3* in all the populations that was significant only for tCD4SP, as well as increased *RUNX1* levels in iCD8SP cells, although these results were not confirmed at the protein level (**Figure 5C** and **Figure S4B**). Finally, we performed unsupervised in-depth clustering analysis of the concatenated flow cytometry data of total thymocytes *ex vivo* and IL-4-exposed CD4SP cells using tSNE dimensional reduction plots (**Figure 5D**). This data representation further showed that cultured CD4SP thymocytes that downregulated ThPOK clustered closely together with *ex vivo* CD8SP cells, while Runx3 was less clearly associated with the acquisition of CD8 expression.

Interestingly, we confirmed that the iCD8SP generated in the presence of IL-7 also presented features associated with CD8SP thymocytes, such as *CD4* and *ThPOK* downregulation and increase in *CD8* and *RUNX3* mRNA levels (**Figure S4C**), despite the very low frequency of coreceptor reversal observed.

In short, our data demonstrate that IL-4 induces clear downregulation of ThPOK in CD4SP thymocytes, both at the mRNA and protein levels. Given that none of the other transcription factors involved in lineage commitment analyzed was consistently modulated by IL-4, our results suggest that ThPOK downregulation is likely the driver of coreceptor reversal in these cells.

## Discussion

CD4^+^ and CD8^+^ T cell development occur in the thymus through a sequence of stages that result in the formation of a functional and diverse repertoire. Here, we show that human CD4SP thymocytes have the potential to become CD8SP cells in the presence of IL-4. IL-4-induced iCD8SP thymocytes are stable upon cytokine removal, in agreement with their sustained ThPOK downregulation, and feature a diverse TCR repertoire. Furthermore, IL-4 also induces an innate-like program on CD4SP and CD8SP thymocytes, resulting in the generation of both conventional and Eomes^+^ innate-like CD8^+^ T cells. Importantly, this ability extends to cord blood CD4 T cells.

During human and murine T cell development DP thymocytes reportedly downregulate CD8 expression, leading to the formation of CD4^+^CD8^low^ cells that can still be converted into the CD8SP lineage upon cytokine signaling (2, 17). In mice the main cytokine involved in this kinetic model is IL-7 (32). Although our sorted CD4SP cells may include some of this intermediate CD4SP CD3^high^ cells, it was surprising to find that IL-4 was able to induce much stronger coreceptor reversal in human CD4SP thymocytes than IL-7. In support of a distinct role for IL-4 in human as compared to murine thymocytes, we showed similar ability of IL-4 and IL-7 to increase Bcl-2 levels in CD4SP cells, suggesting comparable effects on cell survival, in contrast to previous murine data (33). Moreover, in an *in vitro* murine model of positive selection and CD8^+^ T cell differentiation from DP thymocytes in the presence of peptide-coated stromal cells and cytokines, culture supplementation with IL-13 resulted in CD8SP formation (34). We were unable to reveal an effect of IL-13 on CD8 expression in CD4SP thymocytes, probably because these cells lack the appropriate receptor, or due to the lack of concomitant exogenous TCR stimulation. The same study also reported the IL-4-induced generation of Eomes^+^ innate-like CD8^+^ thymocytes in their DP-based murine *in vitro* system, which was not found in cultures supplemented with IL-7, in agreement with our results. Our data support a key role for IL-4 in coreceptor reversal of CD4SP thymocytes, as well as in innate-like T cell development in the human thymus.

We observed coreceptor reversal not only in CD27^neg^ CD4SP cells but also in cells already expressing the maturation and lineage commitment marker CD27, and to lower levels in potentially egressing CD45RA^+^ CD4SP thymocytes. CD8 upregulation on CD4SP thymocytes decreased with maturation stage, though it was not age-dependent in children up to 1 year old, indicating a preserved potential of those cells to generate conventional and/or innate CD8SP cells. Importantly, the diverse repertoire and continuous CD8-upregulating potential of the newly formed CD4SP-derived iCD8SP thymocytes strongly supports that IL-4 is able to induce coreceptor reversal in a broad CD4SP population, and not in a specific population or subset.

Our data showed that cord blood CD4 T cells could also be converted into iCD8SP cells in the presence of IL-4. However, peripheral CD4 T cell plasticity and ability to undergo coreceptor reversal decreased with age (children>adult) and differentiation state (naïve>memory). Although we can infer from our data that this is potentially associated with distinct cell-intrinsic properties, additional studies will be required to determine the basis of the differences observed and their relation to the Th2-skewed immune response that characterizes neonatal T cell immunity (35).

IL-4-induced *de novo* CD8 expression had been described in peripheral CD4 T cell clones cultured with irradiated allogeneic peripheral blood lymphocytes and JY cells in the presence of phytohemagglutinin, although their phenotype relied on continuous cytokine presence (36). In addition, mature intraepithelial CD4^+^ T cells in the mouse intestine have been shown to upregulate CD8αα in response to cues such as TGF-β and retinoic acid (14, 15). However, only CD4^+^CD8^+^ double-positive and not CD4^neg^CD8^+^ single-positive T cells were reported to be generated in those studies, and these cells expressed CD8αα and not CD8αβ, which has much stronger affinity for antigen recognition on MHC class I molecules (30). Other CD4^+^CD8αβ^+^ double-positive T cells do exist at low frequency in the periphery alongside conventional CD4^+^ and CD8^+^ T cells, and they have been linked to conditions such as infections, autoimmune diseases and cancer (37). In cancer settings, CD4^+^CD8^+^ cells were shown to produce IL-4 themselves (37), thus probably promoting the induction of IL-4-related programs. They appear to play an important role at peripheral sites, and some studies have reported their cytotoxic potential, though they may also have suppressive function (37). Whether peripheral CD4^+^CD8αβ^+^ double-positive T cells include a transient population giving rise to CD8^+^ T cells in the presence of IL-4 remains to be determined.

A question that arises from our results is how TCR specificity impacts on CD4-derived CD8 T cells. Antigen-restricted MHC class II-specific CD8^+^ T cells have been described in HIV-infected patients (38–40). Moreover, a recent study reports the *in vivo* generation of a CD4−CD8αβ^+^ MHC II-recognizing lineage from effector CD4^+^ T cells using murine models of acute infection (41). In addition, an increase in thymic innate CD8 thymocytes has recently been shown to be associated with *T. cruzi* murine infection *in vivo*, and those cells were able to protect against lethal infection in an antigen-independent manner (42). In line with these reports, IL-4-induced iCD8SP cells may support an MHC-II-biased or even an MHC-unbiased response to foreign pathogens.

In the thymus, IL-4 has been described to be mainly produced by NKT cells or T-T CD4 thymocytes (27, 43). We directly quantified IL-4 production *ex vivo* in human postnatal thymocytes in the absence of exogenous stimulation and identified, on a per cell basis, CD4SP thymocytes as the strongest IL-4 producers. We also found that a large fraction of low-level IL-4-producing cells are DP CD3^low^ thymocytes, in agreement with a previous report of measurable *IL4* mRNA levels in this population in fetal thymuses (44). Importantly, our histological data showed that IL-4 was mainly expressed in the medullary region in postnatal thymuses, in support of its physiological role in CD4SP thymocyte plasticity. The high IL-4 production by postnatal human CD4SP thymocytes and their elevated IL-4Rα levels argue in favor of a possible feedback loop that can result in a shift of their fate. In agreement, we found an inversion of the CD4/CD8 ratio in thymic tissue supplemented with IL-4, indicating that IL-4 may impact T cell development in situations where IL-4 is systemically increased, such as chronic inflammation (45).

IL-4-mediated conversion of CD4SP thymocytes into stable iCD8SP cells was paralleled by transcriptional and translational downregulation of ThPOK, the master transcription factor for CD4^+^ T cell commitment, which likely constituted the molecular basis for the observed coreceptor reversal. In support of this possibility, ThPOK was shown to directly promote CD8 silencing during murine CD4 lineage commitment, and to suppress CD8 lineage gene expression in peripheral CD4^+^ T cells (13). Moreover, disruption of ThPOK or mutations in its zinc finger domains redirect MHC class II-restricted murine thymocytes into the CD8^+^ lineage, leading to the formation of MHC class II-specific CD8SP cells (6, 7, 46–48). In addition, post-thymic loss of ThPOK derepresses the CTL program in mature CD4 T cells in mouse blood and peripheral tissues under inflammatory conditions (14). Since ThPOK also negatively regulates RUNX3 expression, a crucial factor for full commitment to the cytotoxic CD8 T cell lineage, it could be expected that IL-4 would induce an increase in RUNX3 expression concomitantly with the ThPOK downregulation (46). However, we did not find substantial differences in RUNX3 mRNA or protein levels upon culture with IL-4. Importantly, we noticed that *ex vivo* CD4SP thymocytes already expressed RUNX3, while *ex vivo* CD8SP thymocytes completely lacked ThPOK expression. This was in agreement with previous whole-genome sequencing data of human thymocytes, where *ThPOK* mRNA was only detected in CD4SP while *RUNX3* mRNA was present both in CD8SP and CD4SP cells (49). We thus hypothesize that IL-4-induced ThPOK downregulation on CD4SP cells was enough to revert their fate and induce a cytotoxic program due to the basal expression of RUNX3 by CD4SP thymocytes, which may guarantee an epigenetic “poised” state that allows coreceptor reversal of this population. The mechanisms by which IL-4 induces ThPOK downregulation and CD8 expression in CD4SP thymocytes, including STAT6-dependent and independent signaling pathways and epigenetic regulation, are currently under investigation.

IL-4 has been described to promote Eomes expression on CD8^+^ T cells and to be required for its maintenance in thymic innate-like and peripheral memory CD8^+^ T cells in mice (22, 27, 50). We show here that IL-4 induces not only the formation of conventional CD8SP thymocytes from human postnatal CD4SP thymocytes, but also the emergence of Eomes^+^T-bet^neg^ innate-like iCD8SP thymocytes, which are very similar to those derived from paired CD8SP thymocytes since they also express high levels of innate-associated markers, such as CXCR3 and CD44. We further extended these results to the periphery, as cord blood T cells are still able to generate Eomes^+^ cells in response to IL-4, with potential implications for early defense mechanisms in the neonatal period.

In conclusion, we show here that IL-4 modulates the plasticity of human CD4SP thymocytes and cord blood CD4^+^ T cells, promoting their conversion into CD8^+^ T cells, as well as the acquisition of an innate-like profile. This may be particularly important during early childhood, when a fully competent adaptive immune response is yet to be established, and points to novel pathways to modulate anti-viral immunity and tumor immune responses.

## Data Availability Statement

The original contributions presented in the study are included in the article/**Supplementary Material**. The raw data supporting the conclusions of this article will be made available by the authors. Further inquiries can be directed to the corresponding authors.

## Ethics Statement

The study was reviewed and approved by the Ethical Boards of Hospital de Santa Cruz, Faculty of Medicine of the University of Lisbon and CHULN Lisbon, Portugal. Written informed consent to participate in this study was provided by the participants’ legal guardian/next of kin.

## Author Contributions

HN-C and AS designed the study. HN-C, AR-d-S, AP, and LM performed research and analyzed the data. HN-C, JB, and AS discussed the results. HN-C and AS wrote the paper. All authors contributed to the article and approved the submitted version.

## Funding

This work received funding from PAC - PRECISE - LISBOA-01-0145-FEDER-016394, co-funded by FEDER through POR Lisboa 2020 - Programa Operacional Regional de Lisboa PORTUGAL 2020 and Fundação para a Ciência e a Tecnologia (FCT). HN-C and LM received fellowships from FCT.

## Conflict of Interest

The authors declare that the research was conducted in the absence of any commercial or financial relationships that could be construed as a potential conflict of interest.

## Publisher’s Note

All claims expressed in this article are solely those of the authors and do not necessarily represent those of their affiliated organizations, or those of the publisher, the editors and the reviewers. Any product that may be evaluated in this article, or claim that may be made by its manufacturer, is not guaranteed or endorsed by the publisher.
