# Supplementary material for: Human CD4 T Cells From Thymus and Cord Blood Are Convertible Into CD8 T Cells by IL-4
Source: Front Immunol. 2022 Feb 11;13:834033. doi: 10.3389/fimmu.2022.834033 (PMC8880616; doi:10.3389/fimmu.2022.834033)
Supplement: Supplementary file 1 [file DataSheet_1.pdf]

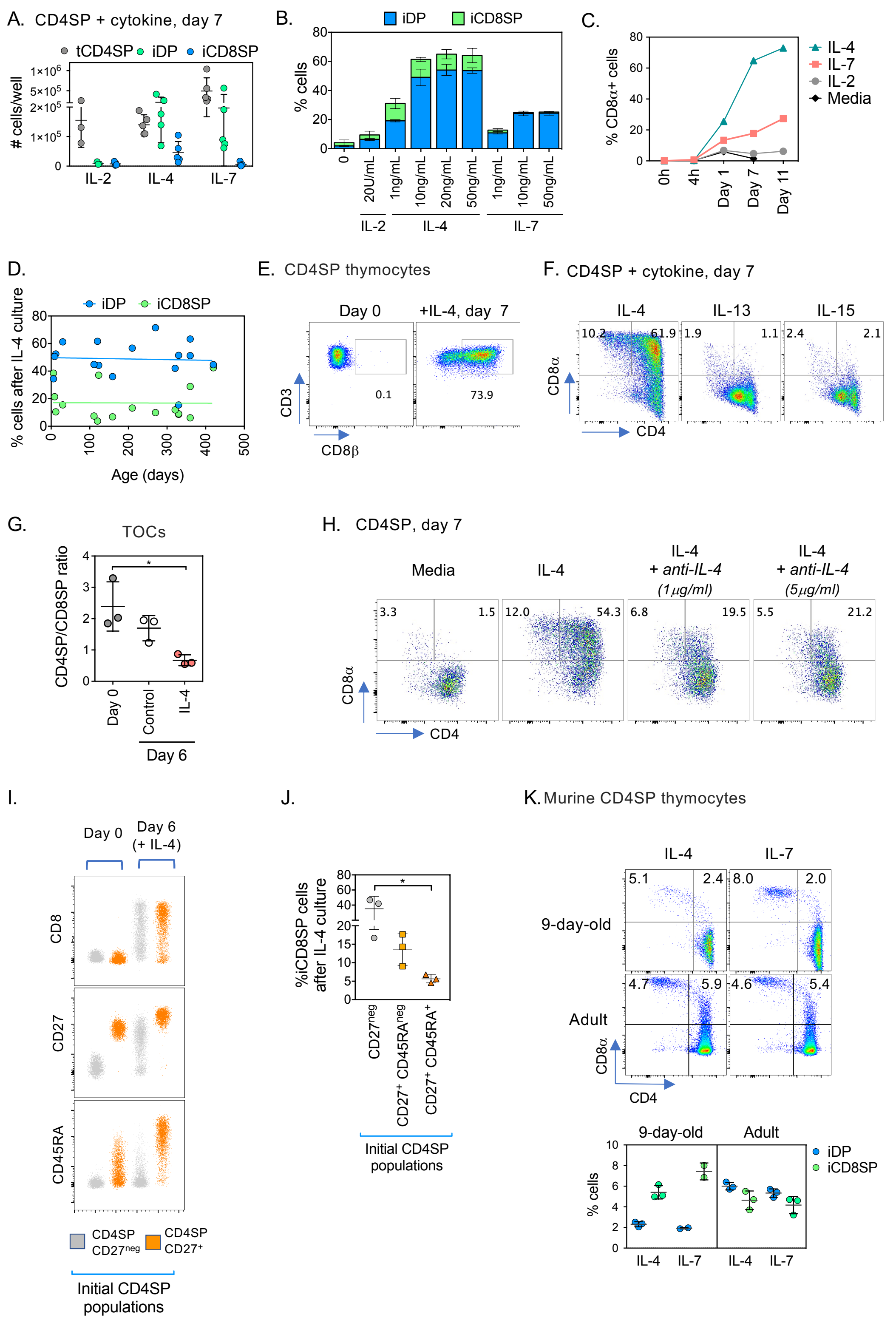

Fig. S1: IL-4 induces the upregulation of CD8αβ on human CD4SP thymocytes. (A) Number of cells per well after 7 days of culture of purified CD4SP thymocytes with IL-2, IL-4 or IL-7. (B) Dose-dependent response of CD4SP thymocytes to IL-2, IL-4 or IL-7, measured as the frequency of iDP (blue bars) and iCD8SP (green bars) cells at the end of the 7-day culture (n=2). (C) Kinetics of CD8α expression in CD4SP thymocytes

incubated with IL-2, IL-4 or IL-7 or cultured in medium alone. (D) Frequency of IL-4-induced iDP (blue dots) and iCD8SP (green dots) cells at the end of the 7-day culture as a function of age. Each dot represents a thymus. (E) CD3 and CD8β expression in CD4SP thymocytes before and after IL-4 culture. (F) Expression of CD8α in CD4SP thymocytes cultured with IL-13 or IL-15 for 7 days. (G) CD4SP/CD8SP ratio in thymic organ cultures (TOCs), on day 0 and after 6 days of culture in medium only (control) or in presence of IL-4. (H) Expression of CD4 and CD8α on CD4SP thymocytes cultured for 7 days with IL-4 in the absence or presence of neutralizing anti-IL-4 antibody at the concentrations mentioned. (I) Expression of CD8, CD27 and CD45RA on CD4SP $CD27^{neg}$ or $CD27^{+}$ sorted cells before and after IL-4 culture. (J) Frequency of iCD8SP thymocytes generated from CD4SP $CD27^{neg}$, $CD27^{+}CD45RA^{neg}$ and $CD27^{+}CD45RA^{+}$ thymocytes after 7 days with IL-4. (K) CD4 and CD8 expression on sorted murine CD4SP thymocytes after 6 days of culture with IL-4 or IL-7. Results in graphs are presented as mean±SD.

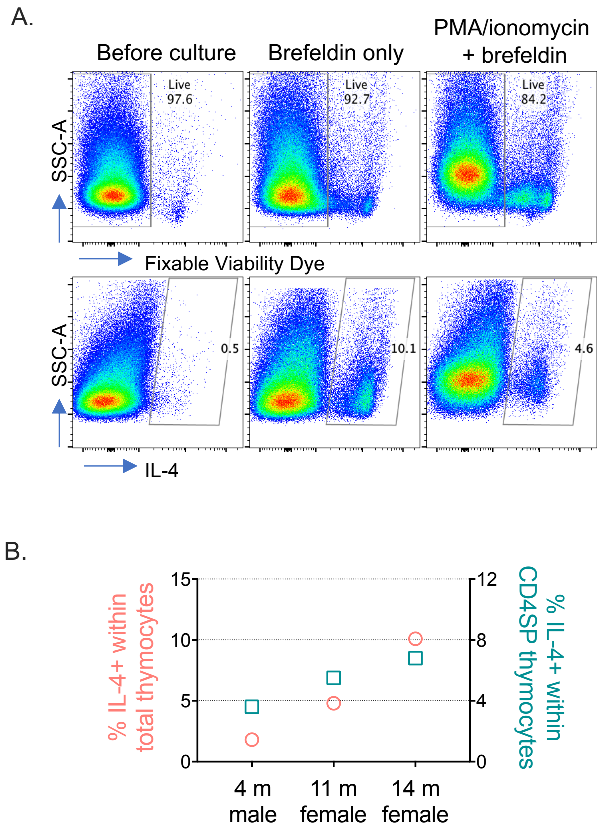

Fig. S2: IL-4 expression in the thymus. (A) Comparison of the frequency of live cells (top dot plots) and of IL-4^+ cells (bottom dot plots) before culture, after 4h of incubation with brefeldin or after 4h of stimulation with PMA/ionomycin plus brefeldin. (B) Frequency of IL-4^+ cells in pediatric thymuses.

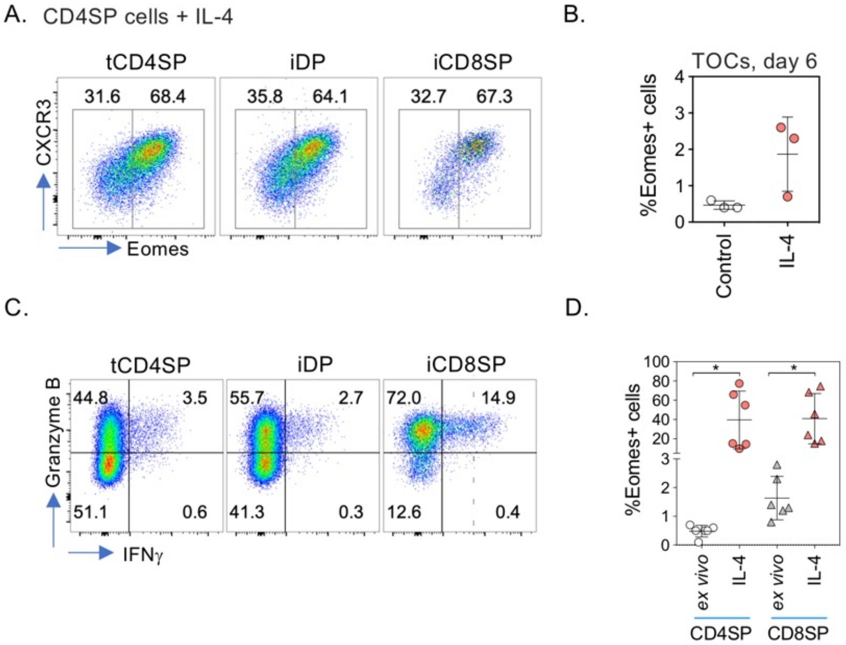

Fig. S3: IL-4 induces an innate-like phenotype on CD4SP thymocytes. (A) Expression of Eomes and CXCR3 in tCD4SP, iDP and iCD8SP cells after 7 days of exposure to IL-4. (B) Frequency of Eomes^{+} cells in thymic organ cultures (TOCs) after 6 days of culture in the absence or presence of IL-4. (C) Representative dot-plots of granzyme B and IFN-γ expression in tCD4SP, iDP and iCD8SP, sorted 7 days after IL-4 treatment, upon 5 days of TCR stimulation and 4h of culture with PMA/ionomycin. (D) Frequency of Eomes-expressing cells before and after IL-4 culture. Results in graphs are presented as mean±SD.

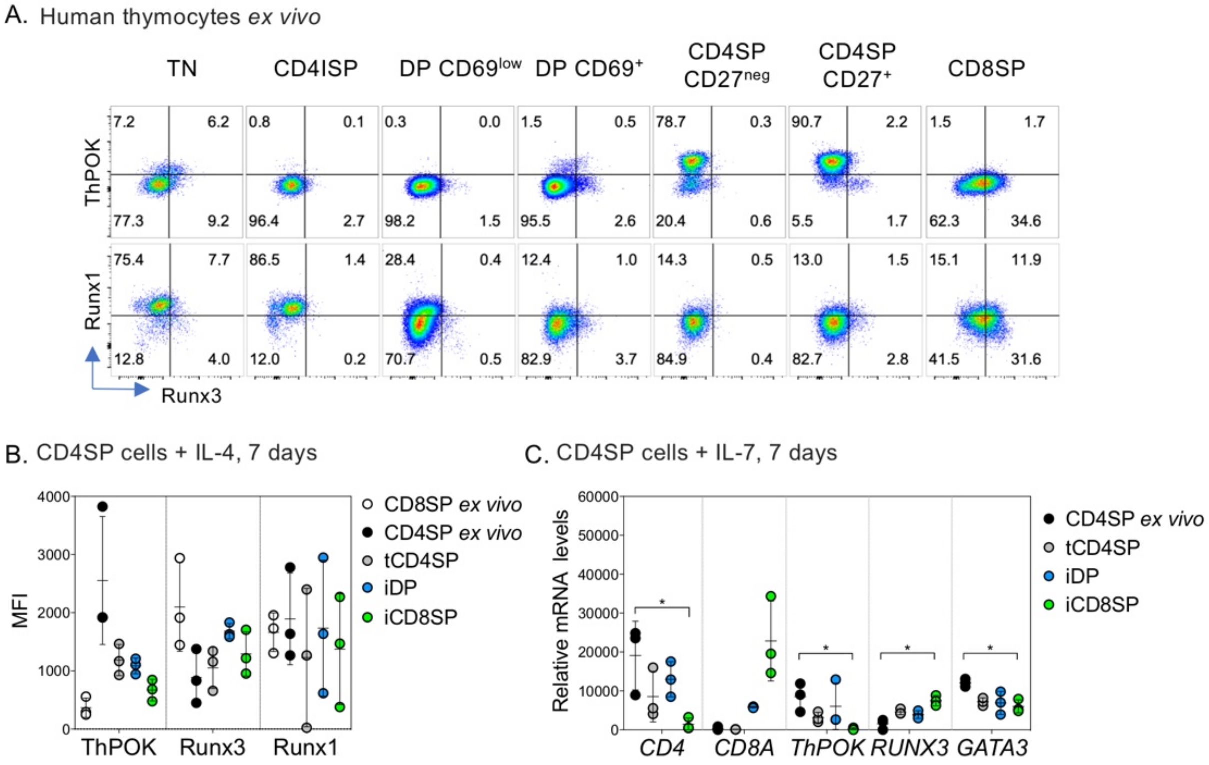

Fig. S4: Characterization of transcription factor modulation in the human thymus and following IL-4 exposure. (A) Representative dot plots of ThPOK, Runx3 and Runx1 protein expression during human T cell developmental stages (3-month-old baby girl). (B) ThPOK, Runx3 and Runx1 median fluorescence intensity (MFI) quantification in sorted CD4SP and CD8SP populations *ex vivo* and in IL-4-induced populations 7 days after culture of CD4SP thymocytes. (C) Expression of *CD4*, *CD8A*, *ThPOK*, *RUNX3* and *GATA3* mRNA levels in CD4SP thymocytes *ex vivo* and in sorted IL-7-induced populations 7 days after culture. Results in graphs are presented as mean±SD.
